# Use of e-Cigarettes and Attendance at Stop Smoking Services: A Population Survey in England

**DOI:** 10.3390/toxics10100593

**Published:** 2022-10-07

**Authors:** Greg Hartwell, Matt Egan, Jamie Brown, Triantafyllos Pliakas, Mark Petticrew

**Affiliations:** 1 Department of Public Health, Environments & Society, London School of Hygiene & Tropical Medicine, London WC1H 9SH, UK; 2 Health Behaviour Research Centre, University College London, London WC1E 6BT, UK; 3 Impact Epilysis, Taxiarchon 35, Kalamaria, 55 132 Thessaloniki, Greece

**Keywords:** electronic cigarettes, e-cigarettes, smoking, tobacco, addiction, addictive behavior, health services, access to healthcare

## Abstract

Little is known about whether e-cigarette use influences tobacco smokers’ decisions around other smoking cessation options, including the most effective one available: stop smoking service (SSS) attendance. Our repeat cross-sectional survey therefore assessed associations between use of e-cigarettes with past and planned future uptake of SSSs. Nicotine replacement therapy (NRT) use was also assessed as a comparator. Participants were drawn from the Smoking Toolkit Study, a nationally representative, validated, face-to-face survey. Data were aggregated on 2139 English adults reporting current smoking of cigarettes or other tobacco products. Multivariable logistic regression was used to adjust for potential confounders. Results showed dual users of combustible tobacco and e-cigarettes were more likely than other smokers to report having accessed SSSs in the past (AOR 1.43, 95% CI 1.08 to 1.90) and intending to take up these services in future (AOR 1.51, 95% CI 1.14 to 2.00). Dual users of combustible tobacco and NRT showed similar associations. Secondary objectives provided evidence on key psychosocial factors that influenced smokers’ decision-making in this area. In summary, despite speculation that e-cigarette use might deter smokers from accessing SSSs, our study found dual users of tobacco and e-cigarettes were more likely to report uptake of such services, compared to smokers not using e-cigarettes.

## 1. Introduction

The last decade has seen major shifts in smokers’ behaviours relating to nicotine consumption and smoking cessation. Behavioural counselling, for instance (the most effective route known for quitting smoking when combined with licensed pharmacotherapy) [1,2], has experienced sustained declines in uptake. In England, the stop smoking services (SSSs) that provide such support to smokers have seen attendance rates drop year-on-year for almost a full decade [3], a decline mirrored in equivalent services across the EU [4]. Over a similar timeframe, the prevalence of regular e-cigarette use has increased in the UK from an estimated 700,000 people in 2012 to an estimated 3.6 million in 2021 [5], and it has been suggested these diverging trends in use of e-cigarettes and SSSs may be linked [6,7,8]. In other words, declines in service uptake could be related to increases in vaping prevalence. This hypothesis is the subject of recurrent debate given its important public health implications; after all, if e-cigarettes suppress uptake of behavioural support, this may exacerbate smoking-related health inequalities (SSSs are notably effective at supporting smokers from lower socioeconomic groups to quit) [1,9]. Similarly, although there is a growing evidence base about the relative level of effectiveness of using e-cigarettes in a smoking cessation attempt [10,11,12,13], researchers and policy-makers remain keen to monitor connected issues with potential public health impacts. Growing research has focused, for instance, on the prevalence of vaping amongst non-smoking adolescents, potential health harms posed by long-term use of e-cigarettes, or support for ex-smokers to quit ongoing vaping.

The role that e-cigarettes play within the smoking cessation sector thus remains highly topical and subject to wide differences internationally in terms of policy, guidance and regulations [14]. In England, SSSs are not permitted to prescribe e-cigarettes, so do not offer them to clients in the same way that they currently provide behavioural support and access to NRT as part of their standard provision. The English National Institute for Health and Care Excellence (NICE) recommends professionals advise that e-cigarettes, while substantially less harmful than smoking, are not risk-free [15]. Specific guidance for SSSs issued by key professional organisations has recognised that behavioural support is most crucial for improving odds of quitting and has recommended SSS practitioners can work with smokers who wish to use their own e-cigarettes alongside SSS support [10,16,17]. Yet, among smokers who vape, SSS attendance rates have been far lower than amongst other smokers [18]. Some smokers who would otherwise have accessed SSSs may therefore be choosing to try quitting through the less effective route of vaping alone, either out of personal preference or due to local services being reduced. Several councils have even posited the popularity of e-cigarettes as part of a rationale for decommissioning local SSSs entirely [19,20,21].

Qualitative studies in this area suggest that smokers, particularly from disadvantaged backgrounds, are influenced by both internal and external factors when deciding whether to attend SSSs [22,23,24]. Beliefs about the effectiveness of SSSs appear particularly influential, as well as fears about how smokers will be received or welcomed by the services (including their expectations of being judged by practitioners, for instance). Meanwhile, qualitative research on e-cigarettes has generally studied them in isolation from other quit methods. Little is known, for instance, about how smokers’ knowledge and beliefs about vaping could relate to their decision-making around other smoking cessation options.

Similarly, research has only recently begun to explore whether vaping amongst smokers may be specifically affecting behavioural support uptake, with mixed findings. A recent UK prospective study suggested that, amongst smokers making a “serious quit attempt”, use of e-cigarettes was associated with reduced likelihood of specifically using behavioural support or prescription medication [25]. Although conclusions that can be drawn from cross-sectional or ecological research are more limited, available studies have found different results. An earlier UK time series analysis found no clear evidence for population-level associations between e-cigarette use and behavioural support uptake [26]. A cross-sectional US survey meanwhile suggested that amongst dual users of combustible tobacco and e-cigarettes almost all age groups were as likely to access such support as other smokers [27].

None of these studies were designed to assess possible sociodemographic interactions, however, or mechanistic associations with related knowledge and beliefs. In fact, no studies outside the US have examined sociodemographic differences in behavioural support uptake amongst smokers using e-cigarettes. Furthermore, no studies anywhere have examined such smokers’ intended future SSS use—a variable with clear implications for the long-term viability of these particularly effective services—or to control for important beliefs and knowledge that could also influence service uptake. Our study therefore aimed to examine whether e-cigarette use (and NRT use as a comparator) were associated with past and planned SSS uptake among smokers. Secondary objectives were to explore potential sociodemographic differences in these outcomes, as well as the kinds of knowledge and beliefs about e-cigarettes and SSSs that were associated with them.

## 2. Methods

### 2.1. Design

This repeat cross-sectional study’s data were collected through the Smoking Toolkit Study (STS), a monthly survey dating back to 2006 [28]. STS sampling is a hybrid between random location and quota: small output areas of approximately 200 households are stratified by geodemographic ordering of the population and randomly selected. Trained interviewers are assigned pre-specified quotas to fulfil, tailored to the areas, before undertaking face-to-face interviews with single members of households. Recruitment is from the general population, with each monthly dataset involving approximately 1700 adults (16+). Previous research demonstrates the STS’s national representativeness [28].

### 2.2. Study Population

This research was approved by the appropriate ethics committees (see ‘Institutional Review Board Statement’) and conformed to the principles embodied in the Declaration of Helsinki. Data were collected between February and November 2017 from 13,735 English adults, with each monthly dataset providing a unique sample of individuals (no repeat interviews occurred). The study sample was created from those 2313 respondents, pooled from the multiple months, who responded to the question “Which of the following best applies to you?” by selecting either “I smoke cigarettes (including hand-rolled) every day”, “I smoke cigarettes (including hand-rolled), but not every day” or “I do not smoke cigarettes at all, but I do smoke tobacco of some kind (e.g., pipe, cigar or shisha)”.

### 2.3. Measures

#### 2.3.1. Measurement of e-Cigarette/NRT Use

All questions and response options are detailed in the study’s questionnaire (Supplementary Material S1). Existing STS questions provided data on current use of e-cigarettes and/or NRT. As with previous studies incorporating STS data [26,29,30], these concepts were measured via three separate questions to capture all relevant smokers and maximise accuracy (“Do you regularly use any of the following in situations when you are not allowed to smoke?”, “Are you using any of the following either to help you stop smoking, to help you cut down or for any other reason at all?”, “Which, if any, of the following are you currently using to help you cut down the amount you smoke?”). Current e-cigarette use was defined as selecting ‘Electronic cigarette’ from the possible responses to any of these questions, with current NRT use defined as choosing any of the nicotine products listed: nicotine gum, nicotine lozenge, nicotine patch, nicotine inhaler\inhalator, another nicotine product or nicotine mouthspray. Respondents selected multiple products if relevant.

#### 2.3.2. Measurement of Outcomes

Primary outcome variables were previous SSS use (‘past uptake’) and future intention to access services (‘planned uptake’), measured by asking “Have you ever sought help from an NHS stop smoking service at any point in the past?” and “How likely or unlikely are you to consider seeking help from your NHS stop smoking service at any point in the future?”. The latter was a single-item measure with five response options; for analysis and interpretation, data were dichotomised to reflect any intention to access services (“Very likely” or “Fairly likely”) versus no intention (“Very unlikely”, “Fairly unlikely” or “Neither likely nor unlikely”).

#### 2.3.3. Measurement of Potential Confounders

Our analysis plan specified confounders a priori, with the exception of two sensitivity analyses outlined below. Existing STS questions provided data on sociodemographics and smoking-related factors. Sociodemographics included age, gender, ethnicity (dichotomised into white versus non-white) and social grade (dichotomised into ABC1 versus C2DE). The established ‘Motivation To Stop Scale’ (MTSS) recorded intention to quit smoking (“Which of the following best describes you?”, dichotomised into “I REALLY want to stop smoking and intend to in the next month”, “I REALLY want to stop smoking and intend to in the next 3 months” or “I want to stop smoking and hope to soon” versus “I REALLY want to stop smoking but I don’t know when I will”, “I want to stop smoking but haven’t thought about when”, “I think I should stop smoking but don’t really want to” or “I don’t want to stop smoking”) [31]. The established ‘Heaviness of Smoking Index’ (HSI) assessed nicotine dependence [32]. Past year quit attempts were assessed by asking “How many serious attempts to stop smoking have you made in the last 12 months?” (dichotomised into zero attempts versus 1+ attempts).

Data were also collected on knowledge and beliefs that could potentially influence SSS attendance or e-cigarette use. Participants were asked: “To what extent do you agree or disagree with each of the following statements?”. Statements covered potential facilitators and barriers to uptake of the respective quit methods, including perceived ease of use/access and reporting of peer precedents who had tried them (see Supplementary Material S1 for comprehensive list of statements).

Responses, based on five-point Likert scales, were dichotomised into “Strongly agree” or “Tend to agree” versus “Neither agree nor disagree”, “Tend to disagree” or “Strongly disagree”. Responses to the question “Out of these two approaches for quitting smoking, which do you think would be more likely to help someone to quit?” were dichotomised into “Getting support from NHS SSSs” versus “Using e-cigarettes” or “Both equally likely”. Finally, participants reporting previous SSS uptake were asked “Overall, to what extent did you find the NHS SSS you attended helpful or not for your efforts to quit smoking?” (responses dichotomised into “Very helpful” or “Fairly helpful”, versus “Not very helpful” or “Not at all helpful”).

### 2.4. Testing of Questions

Seventeen members of the public with varied experiences of smoking, using e-cigarettes/NRT and accessing SSSs were recruited purposively at the research’s outset for face validity testing of the new survey questions proposed. These people reviewed draft questions by email and provided written feedback on their overall merits, as well as any specific wording within them that could be clearer. Seven subject matter experts (tobacco researchers, national policy-makers, survey specialists and SSS staff) were consulted in the same way.

### 2.5. Statistical Analyses

Our planned analyses and sample size calculation were pre-registered publicly on Open Science Framework (www.osf.io/ur3j8, accessed on 30 August 2022). Descriptive statistics were produced for sociodemographic and smoking-related variables, with chi-squared and t tests undertaken to examine potential differences in these by use of e-cigarettes or NRT (Table 1). Final analyses investigated the impact of dual use (of combustible tobacco and e-cigarettes or NRT respectively) on SSS uptake (past or planned respectively), adjusting for smoking-related and sociodemographic co-variables. These furthermore assessed interactions between the dual use variables and key sociodemographics (age, gender, social grade, ethnicity) on past or planned SSS uptake.

Analyses were structured as follows. First, multivariable logistic regression models (M1) were produced for exploratory analyses of knowledge and beliefs concerning e-cigarettes and SSSs. These examined the impact of each knowledge/belief variable in turn on SSS uptake (past and planned respectively), after adjusting for smoking-related and demographic co-variables. Secondly, we developed unadjusted logistic regression models (M2) examining the impact of the dual use variables on the SSS uptake variables to provide crude odds ratios (ORs). Thirdly, we developed fully adjusted models (M3) examining the impact of each dual use variable in turn on each SSS uptake variable, after adjusting for a priori variables and statistically significant knowledge/belief variables (*p* < 0.05) identified in M1, in order to produce final adjusted ORs with 95% CIs. In a further stage, we also examined interactions between each dual use variable and key sociodemographic variable (socioeconomic status, age, gender, ethnicity) on each SSS uptake variable. This involved developing a series of different ‘interaction’ models—each model having the interaction term in question (e.g., dual use of combustible tobacco and e-cigarettes x gender)—which adjusted for all a priori and other statistically significant variables (as in M3). Following these pre-registered analyses, some unplanned sensitivity analyses explored, in the M3 models, the impact of including two potentially relevant further variables: use of NRT (when examining dual combustible tobacco/e-cigarette use) or e-cigarettes (when examining dual combustible tobacco/NRT use), as well as past SSS uptake (when examining planned SSS uptake). Analyses were undertaken using SPSS v24.

## 3. Results

### 3.1. Sample Characteristics

Out of 2313 smokers interviewed, complete data on key co-variables (HSI, age and gender) was provided by 2189 (94.5%). Those excluded due to missing data (5.0% HSI, 0.4% age, 0.1% gender) were significantly less likely to be white or female (*p* < 0.05) than those remaining. Both groups of dual users were likelier than other smokers to report a quit smoking attempt within the previous year and a future quit intention. Dual users of combustible tobacco/e-cigarettes were similar to other smokers in most sociodemographic characteristics (Table 1), but were significantly likelier to be white or Northern England residents. Dual users of combustible tobacco/NRT were significantly older than other smokers and likelier to have a disability or to be Southern England residents, but less likely to be heterosexual or Northern England residents.

18.2% of participants (399/2189) were currently using e-cigarettes, 10.2% (223/2189) were using NRT and 74.1% were using neither (1622/2189). 21.6% of participants (472/2189) had accessed SSSs previously and 23.2% (508/2189) planned to do so in future.

### 3.2. Knowledge and Beliefs Regarding e-Cigarettes and SSSs (M1)

In the M1 analyses of knowledge and belief variables (see Supplementary Material S2 for comprehensive findings), having accessed SSSs in the past and planning to do so in future were associated with knowing people who used e-cigarettes (AOR = 1.79, 95% CI: 1.35–2.38 for past uptake and AOR = 1.43, 95% CI: 1.09–1.88 for planned uptake) and thinking that e-cigarettes were less effective than SSSs (AOR = 1.33, 95% CI: 1.06–1.65 for past uptake and AOR = 2.35, 95% CI: 1.89–2.93 for planned uptake). Past use of SSSs was also associated with knowing how to use e-cigarettes (AOR = 2.01, 95% CI: 1.54–2.63).

Furthermore, having accessed SSSs in the past and planning to do so in future were associated with: knowing people who had used SSSs (AOR = 3.39, 95% CI: 2.71–4.24 for past uptake and AOR = 1.59, 95% CI: 1.27–1.99 for planned uptake); thinking that SSSs were a convenient way to quit smoking (AOR = 1.73, 95% CI: 1.39–2.16 for past uptake and AOR = 3.07, 95% CI: 2.43–3.87 for planned uptake); knowing how to access SSSs (AOR = 4.66, 95% CI: 3.25–6.69 for past uptake and AOR = 2.00, 95% CI: 1.49–2.68 for planned uptake); and thinking they would be made to feel welcome by SSSs (AOR = 1.99, 95% CI: 1.53–2.58 for past uptake and AOR = 2.91, 95% CI: 2.19–3.87 for planned uptake). Planned uptake was also associated with having found past use of SSSs helpful (AOR = 5.61, 95% CI: 3.57–8.82); thinking dual users of e-cigarettes and combustible tobacco were eligible for SSSs (AOR = 1.32, 95% CI: 1.06–1.63); and thinking lots of time was needed to access SSSs (AOR = 0.61, 95% CI: 0.47–0.79; NB: inversely associated, unlike the others).

### 3.3. Past and Planned Uptake of SSSs (M2&3)

In the M2 unadjusted analyses (Table 2 and Table 3), dual users of combustible tobacco/e-cigarettes were more likely than other smokers to report past (OR 1.93, 95% CI: 1.51–2.45) and planned SSS uptake (OR 1.53, 95% CI: 1.20–1.95). Dual users of combustible tobacco/NRT were also more likely than other smokers to report past (OR 2.93, 95% CI: 2.20–3.91) and planned SSS uptake (OR 3.04, 95% CI: 2.28–4.04). After adjustment for demographic, smoking-related, and knowledge/belief variables in M3, these associations all remained statistically significant (Table 2 and Table 3).

There were no interactions between use and social grade, age or ethnicity for any outcomes. A significant interaction was observed for gender with dual combustible tobacco/NRT use on planned SSS uptake. For females, dual combustible tobacco/NRT use was associated with significantly increased odds of intending to access SSSs (OR 3.40, 95% CI: 2.19–5.28), which was not observed with males (OR 1.45, 95% CI: 0.90–2.35). Similar gender interactions were not evident with other outcomes. In sensitivity analyses further adjusted for NRT use, e-cigarette use or past SSS uptake, results were very similar: dual combustible tobacco/e-cigarette users remained likelier than other smokers to have accessed SSSs previously (AOR 1.43, 95% CI: 1.08–1.91) and to plan future uptake (AOR 1.40, 95% CI: 1.05–1.88), as did dual combustible tobacco/NRT users (past SSS uptake: AOR 2.10, 95% CI: 1.51–2.93; planned uptake: AOR 2.03, 95% CI: 1.45–2.84).

## 4. Discussion

Amongst current smokers, those also using either e-cigarettes or NRT were more likely to report having accessed SSSs in the past and intending to access services in future. To our knowledge, this research is the first of its kind to combine data on e-cigarette use with data about both past and planned behavioural support uptake. It therefore has particular relevance to current debates around the popularity of e-cigarettes and their potential impact on smokers’ decisions regarding cessation services. Another key strength is its use of a representative sample of the English population. Through our secondary objectives, we also generated evidence on what knowledge and beliefs influence smokers when deciding whether or not to access behavioural support, the most effective route available to quitting smoking.

Limitations of our study include the need for some caution when generalising our findings to other populations. Many countries regulate e-cigarettes differently to England, while models of behavioural support available to smokers also vary internationally [14]. Although cross-sectional associations can still be indicative and important for guiding future research, they need to be interpreted with caution given the potential for biases and unknown confounders. For example, we relied—in part—on data gathered using novel questions as there were no relevant established questionnaires from which to take our new questions regarding SSS uptake (though face validity was tested beforehand with a range of smokers reporting varying uptake of different quit routes). It is thus possible that our finding of a positive association between the different dual use variables and planned SSS uptake reflects residual confounding—e.g., it may be caused by smokers’ general motivation to quit smoking more than anything particularly related to SSSs, or by other unidentified confounders. The ‘intention to quit’ concept was, however, captured by the MTSS—an established, validated tool used regularly for broader published analyses of STS data—and was also adjusted for within all our analyses [26,29,30,31]. Finally, social desirability bias may have influenced reported future actions. Larger studies could attempt to tackle this by following up respondents over time and assessing how far intentions to access services translate into genuine uptake. Similarly, sociodemographic differences in choice of quit routes, including behavioural support, remain a valuable area for further research.

This study nonetheless provides important new evidence in an area—associations between e-cigarette use and behavioural support uptake—where a clear understanding has yet to be established. Our findings suggest a modest positive association, with smokers using e-cigarettes or NRT significantly more likely than other smokers to have accessed services previously and to plan future use of them. A plausible explanation is that, given most smokers using e-cigarettes or NRT do so in an attempt to quit smoking [12], the increased reports of past and planned SSS uptake among these groups may reflect willingness to consider other quit methods beyond e-cigarettes/NRT. It also likely reflects that some previous SSS attenders will have been introduced to e-cigarettes or NRT by services directly, and given advice by practitioners, leading to more sustained use of such products compared to non-attenders. Indeed, further research could usefully examine how often such e-cigarette use following English SSS attendance is continuing long-term, given the conclusion of a recent systematic review in this area that “use of e-cigarettes as a therapeutic intervention for smoking cessation may lead to permanent nicotine dependence” [33]. Future intentions to access services in current users of e-cigarettes or NRT may similarly reflect at least in part the fact that some of these smokers will have been introduced to these products through previous use of such services. Cross-sectional research is inevitably limited in conclusions it can draw regarding the temporal or causal nature of such relationships. Our sensitivity analyses did however adjust for past use of services when examining future use (as outlined in ‘Results’), with very similar results to main analyses. Alternatively, experiences with other satisfying nicotine products may stimulate thoughts about quitting and boost self-efficacy. Finally, this phenomenon may link to financial considerations. In numerous studies, smokers report lower costs of e-cigarettes, compared to combustible cigarettes, as a major incentive for use, while it has also been shown that subsidised NRT offered by SSSs is positively associated with quit attempts [34,35]. It is thus plausible that smokers motivated to attempt switching from combustible tobacco to e-cigarettes or NRT for economic reasons may be attracted to this SSS offer of subsidised pharmacotherapy. Our findings align with some aforementioned studies that have not found e-cigarette use to be associated with depressed uptake of behavioural support [26,27]. Conversely, an English study found in an unplanned analysis that dual users of tobacco/e-cigarettes were significantly less likely than dual users of tobacco/NRT to specifically use behavioural support or prescription medication, though the two groups did not differ in their overall use of evidence-based cessation aids [25]. This mixed evidence base could result from differences in study designs, since Beard et al. employed a prospective cohort design [25]. Alternatively, it could reflect the fact that this previous study combined prescription medication with behavioural support, whereas our own isolated the latter. Either way, further studies in other settings directly comparing dual e-cigarette/tobacco use against dual tobacco/NRT use would be valuable given such statistical analyses were not a primary focus of our own. Our study does concur though with Beard et al.’s assertion that a clearer picture in this area requires a greater understanding of the perceptions and motivations of smokers in relation to e-cigarettes and other quit routes.

Our own study provides some further early insights in relation to that specific need, marking an important quantitative contribution to the largely qualitative evidence base on what factors motivate smokers’ choices of quit routes. Despite the earlier caveat regarding the challenges of investigating temporal relationships via cross-sectional research, this study to our knowledge, still constitutes the only quantitative study to date to examine how knowledge and beliefs about e-cigarettes may be influencing uptake of behavioural support. This is particularly salient given the aforementioned ongoing debate as to whether e-cigarettes’ popularity could be depressing uptake of more effective routes to quitting combustible tobacco [4,6,18]. Smokers in our adjusted analyses who reported having acquaintances who used e-cigarettes were more likely to have accessed SSSs in the past and to plan to do so in future, while past SSS use was also associated with reported knowledge of how to use e-cigarettes oneself. This result aligns with recent survey findings that exposure to other people’s e-cigarette use may have some effects on smokers’ quitting motivation and behaviour [36]—perhaps by normalising attempts to quit—as well as with broader research suggesting e-cigarettes are not viewed by smokers as being in competition with, or mutually exclusive from behavioural support [26,27]. Indeed, recent studies have indicated that both current and ex-smoking vapers have an appetite to access other forms of treatment such as behavioural support [37,38]. Our findings further show that reported knowledge and beliefs about vaping have significant associations with planned SSS uptake, including the perception that dual users of e-cigarettes and tobacco are eligible for SSS support. Future research could therefore consider exploring whether or not changing these beliefs about eligibility for SSSs—for instance, through the provision of clearer information to the public about SSS eligibility criteria—may potentially influence intentions to access these services. Similarly, further studies could consider investigating whether or not social connections with other vapers potentially influence knowledge of different quit routes and normalise quitting behaviour, perhaps through discussions with these friend and family ‘precedents’.

## 5. Conclusions

Our study has clear relevance for ongoing debates about the relationship between e-cigarette use, NRT use and the uptake and provision of other quit methods including behavioural support. It has been suggested, for instance, that widespread e-cigarette use may be reducing the need for SSSs, an argument that has formed part of the rationale for cutting such services in a number of English local authorities [19,20,21]. Our findings do not support this argument; rather than wanting to ‘go it alone’, a proportion of smokers in our sample remained keen to receive additional support to quit from SSSs even when already using e-cigarettes. Instead of assuming that long-term declines in SSS attendance are primarily linked to e-cigarette use, alternative explanations should thus also be considered. Future research should explore, for example, the potential role that may be being played by significant cuts in recent years to the local authority public health budgets that fund such services.

## Figures and Tables

**Table 1:** Sample characteristics by dual use of combustible tobacco/e-cigarettes or combustible tobacco/NRT.

	All	Dual e-Cig/Tobacco Use		*p* *	Dual NRT/Tobacco Use		*p* *	Tobacco Use Only		*p* *
	Smokers	Yes	No		Yes	No		Yes	No	
All smokers	-	18.2%	81.8%	<0.001 *	10.2%	89.8%	<0.001 *	74.1%	25.9%	<0.001 *
Demographic characteristics										
Age, Mean (SD)	43.5 (17.3)	43.0 (16.5)	43.6 (17.5)	0.555	47.0 (16.9)	43.1 (17.3)	0.001 *	43.1 (17.4)	44.6 (16.9)	0.086
Female	49.7%	50.9%	49.4%	0.590	54.3%	49.1%	0.147	48.6%	52.6%	0.109
White	90.0%	93.2%	89.3%	0.019 *	90.1%	90.0%	0.961	89.1%	92.6%	0.018 *
Social grade C2DE	56.7%	54.6%	57.2%	0.359	54.3%	57.0%	0.439	57.3%	55.0%	0.352
No 16+ qualifications	60.9%	61.7%	60.8%	0.747	60.5%	61.0%	0.896	60.6%	61.9%	0.585
With disability	17.4%	18.9%	17.1%	0.381	22.4%	16.9%	0.038 *	16.5%	20.0%	0.058
Heterosexual	87.4%	89.4%	87.0%	0.191	82.5%	88.0%	0.020 *	87.6%	86.9%	0.653
Region: North	32.2%	36.8%	31.1%	0.027 *	25.6%	32.9%	0.026 *	32.2%	32.1%	0.971
Central	29.7%	29.1%	29.8%	0.764	29.1%	29.8%	0.851	29.7%	29.6%	0.969
South	38.1%	34.1%	39.1%	0.065	45.3%	37.3%	0.020 *	38.1%	38.3%	0.943
Smoking characteristics										
Intent to quit smoking	33.1%	51.6%	29.0%	<0.001 *	58.3%	30.3%	<0.001 *	25.9%	53.8%	<0.001 *
Past year quit attempt	29.9%	50.9%	25.2%	<0.001 *	59.2%	26.6%	<0.001 *	21.7%	53.3%	<0.001 *
HSI Index, Mean (SD)	1.72 (1.51)	1.78 (1.43)	1.71 (1.53)	0.382	1.79 (1.49)	1.71 (1.51)	0.484	1.71 (1.52)	1.77 (1.47)	0.374

NRT: nicotine replacement therapy; SD: Standard deviation; C2DE: small employers and own account workers, lower supervisory and technical occupations, semi-routine and routine occupations, never workers and long-term unemployed (ABC1: managerial, professional and intermediate occupations); North: North East, North West, Yorkshire and Humber; Central: East Midlands, West Midlands, East of England; South: London, South East, South West; HSI: Heaviness of Smoking Index (index ranges from 0 to 6: the higher the score, the higher the dependence on nicotine); Tobacco use only: current smokers of combustible tobacco with no current use of e-cigarettes or NRT. *: statistically significant (*p* < 0.05).

**Table 2:** E-cigarette or NRT use and past uptake of SSSs amongst current smokers of combustible tobacco.

		Past Uptake of SSSs		
		% [n]	OR [95% CI]	AOR [95% CI]
Dual e-cig/tobacco use	No	19.3% (346/1790)	1.00	1.00
	Yes	31.6% (126/399)	1.93 (1.51–2.45)	1.43 (1.08–1.90)
Dual NRT/tobacco use	No	19.3% (380/1966)	1.00	1.00
	Yes	41.3% (92/223)	2.93 (2.20–3.91)	2.10 (1.51–2.93)

**Table 3:** E-cigarette or NRT use and planned uptake of SSSs amongst current smokers of combustible tobacco.

		Planned Uptake of SSSs		
		% [n]	OR [95% CI]	AOR [95% CI]
Dual e-cig/tobacco use	No	21.7% (389/1790)	1.00	1.00
	Yes	29.8% (119/399)	1.53 (1.20–1.95)	1.51 (1.14–2.00)
Dual NRT/tobacco use	No	20.8% (409/1966)	1.00	1.00
	Yes	44.4% (99/223)	3.04 (2.28–4.04)	2.30 (1.66–3.18)

## Data Availability

The datasets used and analysed during the current study are available from the corresponding author on reasonable request.

## Supplementary Materials

The following supporting information can be downloaded at: https://www.mdpi.com/article/10.3390/toxics10100593/s1, Supplementary Material S1: full list of questions and response options; Supplementary Material S2: full model 1 results.
