# Supplementary material for: Use of e-Cigarettes and Attendance at Stop Smoking Services: A Population Survey in England
Source: Toxics. 2022 Oct 7;10(10):593. doi: 10.3390/toxics10100593 (PMC9610903; doi:10.3390/toxics10100593)
Supplement: Supplementary file 1 [file toxics-10-00593-s001.zip › toxics-1919896-supplementary.pdf]

toxics

MDPI

Supplementary Materials

Use of e-Cigarettes and Attendance at Stop Smoking Services: A Population Survey in England

Greg Hartwell, Matt Egan, Jamie Brown, Triantafyllos Pliakas and Mark Petticrew

Questionnaire S1: Full list of questions and response options

Q: Which, if any, of the following are you currently using to help you cut down the amount you smoke?

- Nicotine gum
- Nicotine replacement lozenges\tablets
- Nicotine replacement inhaler
- Nicotine replacement nasal spray
- Nicotine patch
- Electronic cigarette
- Nicotine mouthspray
- Heat-not-burn cigarette (e.g. iQOS, heatsticks)
- Other (specify)

Q: Do you regularly use any of the following in situations when you are not allowed to smoke?

- Nicotine gum
- Nicotine lozenge
- Nicotine patch
- Nicotine inhaler\inhalator
- Another nicotine product
- Electronic cigarette
- Nicotine mouthspray
- Heat-not-burn cigarette (e.g. iQOS, heatsticks)
- Other (specify)

Q: Can I check, are you using any of the following either to help you stop smoking, to help you cut down or for any other reason at all?

- Nicotine gum
- Nicotine lozenge
- Nicotine patch
- Nicotine inhaler\inhalator
- Another nicotine product
- Electronic cigarette
- Nicotine mouthspray
- Heat-not-burn cigarette (e.g. iQOS, heatsticks)
- Other (specify)

Q: Which of the following best describes you?

- I REALLY want to stop smoking and intend to in the next month
- I REALLY want to stop smoking and intend to in the next 3 months
- I want to stop smoking and hope to soon
- I REALLY want to stop smoking but I don't know when I will
- I want to stop smoking but haven't thought about when
- I think I should stop smoking but don't really want to
- I don't want to stop smoking

Q: How many cigarettes per day do you usually smoke?

- *[Numerical response]*

Q: How soon after you wake up do you light up?

- Within 5 minutes
- 6 - 30 minutes
- 31 - 60 minutes
- More than 60 minutes

Q: Can I please check, as far as you can recall, whether you have made any serious attempts to stop smoking in the last 12 months or not?

- Yes – I have made at least one serious attempt to stop smoking in the last 12 months
- No – as far as I can recall I have not made any serious attempt to stop smoking in the last 12 months

The next few questions relate to local NHS stop smoking services. Any smokers in England wanting to quit can get help from these services. It involves talking to a trained stop smoking practitioner and provides access to a nicotine product or other stop smoking medicine. GPs can set up appointments for their patients or people can contact the service

themselves. They offer one-to-one counselling\advice\support sessions or stop smoking groups. Please note: for the purposes of these questions, 'smoking' is defined as smoking conventional (i.e. tobacco) cigarettes rather than e-cigarettes.

Q: Have you ever sought help from an NHS stop smoking service at any point in the past?

- Yes, in the last 12 months
- Yes, more than 12 months ago
- No

Q: Overall, to what extent did you find the NHS stop smoking service you attended helpful or not for your efforts to quit smoking?

- Very helpful
- Fairly helpful
- Not very helpful
- Not at all helpful

Q: How likely or unlikely are you to consider seeking help from your NHS stop smoking service at any point in the future?

- Very likely
- Fairly likely
- Neither likely nor unlikely
- Fairly unlikely
- Very unlikely

Q: Do you think NHS stop smoking services currently offer their support to smokers who are using e-cigarettes to try to quit smoking, or not?

- Yes
- No

Q: Out of these two approaches for quitting smoking, which do you think would be more likely to help someone to quit?

- Using e-cigarettes
- Getting support from NHS stop smoking services
- Both equally likely

Q: To what extent do you agree or disagree with each of the following statements?

1. I know people who use e-cigarettes

2. I know people who have attended NHS stop smoking services
3. I think e-cigarettes are a convenient way to quit smoking
4. I think NHS stop smoking services are a convenient way to quit smoking
5. I think learning how to use e-cigarettes takes up a lot of time
6. I think using NHS stop smoking services takes up a lot of time
7. I know how to use e-cigarettes if I want to
8. I know how to access NHS stop smoking services if I want to
9. Most of my family and friends accept my e-cigarette use
10. Most of my family and friends accept my smoking
11. If I was to use NHS stop smoking services, I would have to travel far
12. If I attended an NHS stop smoking service in the future, I think I would be made to feel welcome

[Each statement had the following answer options]

- Strongly agree
- Tend to agree
- Neither agree nor disagree
- Tend to disagree

Strongly disagree

Table S1 Full Model 1 results - Knowledge/beliefs and past or planned SSS uptake amongst current smokers

	**Agreement with knowledge/belief statements**		**Ever use of SSS**			**Planned future use of SSS**		
			% reporting SSS ever use (n)	*OR (95% CI)*	*AOR (95% CI)*	*% reporting SSS planned use (n)*	*OR (95% CI)*	*AOR (95% CI)*
**E-cigarettes**	Knows people who use e-cigs	*No*	14.6% (75/512)	1.00	1.00	16.6% (85/512)	1.00	1.00
		Yes	23.7% (397/1677)	**1.81 (1.38-2.37)**	**1.79 (1.35-2.38)**	25.2% (423/1677)	**1.69 (1.31-2.19)**	**1.43 (1.09-1.88)**
	Thinks e-cigs are convenient way to quit	*No*	21.0% (279/1327)	1.00	1.00	21.4% (284/1327)	1.00	1.00
		Yes	22.4% (193/862)	1.08 (0.88-1.33)	1.03 (0.83-1.28)	26.0% (224/862)	**1.29 (1.06-1.58)**	1.22 (0.98-1.51)
	Thinks lots of time needed to learn to use e-cigs	*No*	21.8% (428/1961)	1.00	1.00	23.2% (454/1961)	1.00	1.00
		Yes	19.3% (44/228)	0.86 (0.61-1.21)	0.77 (0.54-1.11)	23.7% (54/228)	1.03 (0.75-1.42)	1.03 (0.73-1.45)
	Knows how to use e-cigs	*No*	14.3% (91/637)	1.00	1.00	17.7% (113/637)	1.00	1.00
		Yes	24.5% (381/1552)	**1.95 (1.52-2.51)**	**2.01 (1.54-2.63)**	25.5% (395/1552)	**1.58 (1.25-2.00)**	1.28 (1.00-1.65)
	Thinks family & friends accept e-cig use [1]	*No*	28.9% (33/114)	1.00	1.00	35.1% (40/114)	1.00	1.00
		Yes	33.0% (93/282)	1.21 (0.75-1.94)	1.16 (0.70-1.91)	27.7% (78/282)	0.71 (0.44-1.13)	0.67 (0.41-1.10)
**Stop smoking services**	Knows people who have used SSSs	*No*	14.3% (210/1471)	1.00	1.00	18.7% (275/1471)	1.00	1.00
		Yes	36.5% (262/718)	**3.45 (2.79-4.26)**	**3.39 (2.71-4.24)**	32.5% (233/718)	**2.09 (1.70-2.56)**	**1.59 (1.27-1.99)**
	Thinks SSSs are convenient way to quit	*No*	16.4% (170/1037)	1.00	1.00	12.5% (130/1037)	1.00	1.00
		Yes	26.2% (302/1152)	**1.81 (1.47-2.24)**	**1.73 (1.39-2.16)**	32.8% (378/1152)	**3.41 (2.73-4.25)**	**3.07 (2.43-3.87)**
	Thinks lots of time needed to access SSSs	*No*	21.7% (352/1622)	1.00	1.00	24.9% (404/1622)	1.00	1.00
		Yes	21.2% (120/567)	0.97 (0.77-1.22)	0.96 (0.75-1.22)	18.3% (104/567)	**0.68 (0.53-0.86)**	**0.61 (0.47-0.79)**
	Knows how to access SSSs	*No*	6.8% (37/541)	1.00	1.00	12.0% (65/541)	1.00	1.00
		Yes	26.4% (435/1648)	**4.89 (3.44-6.94)**	**4.66 (3.25-6.69)**	26.9% (443/1648)	**2.69 (2.03-3.57)**	**2.00 (1.49-2.68)**

	Thinks family & friends accept smoking	*No*	22.2% (199/896)	1.00	1.00	26.0% (233/896)	1.00	1.00
		Yes	21.1% (273/1293)	0.94 (0.76-1.15)	0.99 (0.79-1.23)	21.3% (275/1293)	**0.77 (0.63-0.94)**	0.86 (0.69-1.07)
	Thinks would have to travel far to use SSSs	*No*	22.1% (419/1899)	1.00	1.00	23.6% (448/1899)	1.00	1.00
		Yes	18.3% (53/290)	0.79 (0.57-1.08)	0.72 (0.52-1.00)	20.7% (60/290)	0.84 (0.62-1.14)	0.89 (0.65-1.23)
	Thinks would be made to feel welcome by SSSs	*No*	14.0% (91/652)	1.00	1.00	10.4% (68/652)	1.00	1.00
		Yes	24.8% (381/1537)	**2.03 (1.58-2.61)**	**1.99 (1.53-2.58)**	28.6% (440/1537)	**3.45 (2.62-4.53)**	**2.91 (2.19-3.87)**
	Found past use of SSSs helpful [2]	*No*	N/A	N/A	N/A	20.8% (36/173)	1.00	1.00
		Yes				60.2% (180/299)	**5.76 (3.73-8.88)**	**5.61 (3.57-8.82)**
	Thinks dual e-cig/tobacco users eligible for SSS	*No*	20.8% (283/1359)	1.00	1.00	20.9% (284/1359)	1.00	1.00
		Yes	22.8% (189/830)	1.12 (0.91-1.38)	1.10 (0.88-1.36)	27.0% (224/830)	**1.40 (1.14-1.71)**	**1.32 (1.06-1.63)**
**E-cigs vs SSSs**	Thinks e-cigs are more effective than SSS	*No*	21.9% (406/1855)	1.00	1.00	25.1% (466/1855)	1.00	1.00
		Yes	19.8% (66/334)	0.88 (0.66-1.18)	0.85 (0.62-1.15)	12.6% (42/334)	**0.43 (0.31-0.60)**	**0.40 (0.28-0.57)**
	Thinks SSSs are more effective than e-cigs	*No*	20.2% (287/1421)	1.00	1.00	17.9% (254/1421)	1.00	1.00
		Yes	24.1% (185/768)	**1.25 (1.02-1.55)**	**1.33 (1.06-1.65)**	33.1% (254/768)	**2.27 (1.85-2.78)**	**2.35 (1.89-2.93)**

[1] n=396, as question only asked to respondents reporting current use of e-cigs; [2] n=472, as question only asked to respondents reporting any ever use of SSS.
